# Supplementary material for: Interaction of biomolecules with anatase, rutile and amorphous TiO2 surfaces: A molecular dynamics study
Source: PLoS One. 2023 Sep 5;18(9):e0289467. doi: 10.1371/journal.pone.0289467 (PMC10479902; doi:10.1371/journal.pone.0289467)
Supplement: S1 File — Short videos representing the interaction of the 6 different biomolecules (KGD, KRSR, LGD, LRSR, RGD and RSR) with the 3 different TiO2 surface (anatase, amorphous and rutile). (DOCX) [file pone.0289467.s001.docx]

All relevant data are within the manuscript and its Supporting Information files. The data underlying the results presented in the study are available from the Zenodo Repository.

<https://doi.org/10.5281/zenodo.8036651>

**S1 File. Peptides on TiO_2_ surfaces videos.** Short videos representing the interaction of the 6 different biomolecules (KGD, KRSR, LGD, LRSR, RGD and RSR) with the 3 different TiO_2_ surface (anatase, amorphous and rutile). (ZIP file)
