# Supplementary material for: Interaction of biomolecules with anatase, rutile and amorphous TiO2 surfaces: A molecular dynamics study
Source: PLoS One. 2023 Sep 5;18(9):e0289467. doi: 10.1371/journal.pone.0289467 (PMC10479902; doi:10.1371/journal.pone.0289467)
Supplement: S2 File — A representative trajectory file of the LRSR peptide and amorphous TiO2 surface MD simulation without the ions and water molecules. (DOCX) [file pone.0289467.s002.docx]

All relevant data are within the manuscript and its Supporting Information files. The data underlying the results presented in the study are available from the Zenodo Repository.

<https://doi.org/10.5281/zenodo.8036651>

**S2 File. LRSR trajectory on amorphous surface.** A representative trajectory file of the LRSR peptide and amorphous TiO_2_ surface MD simulation without the ions and water molecules.
