# Supplementary material for: Interaction of biomolecules with anatase, rutile and amorphous TiO2 surfaces: A molecular dynamics study
Source: PLoS One. 2023 Sep 5;18(9):e0289467. doi: 10.1371/journal.pone.0289467 (PMC10479902; doi:10.1371/journal.pone.0289467)
Supplement: S1 Appendix — (DOCX) [file pone.0289467.s004.docx]

**Appendix**

Here we provide figures and data that enables comparison of all simulations. This data includes all combinations of all tri- and tetrapeptides on all three surface types. Note, that part of this information is already presented in the main text of the article.

**
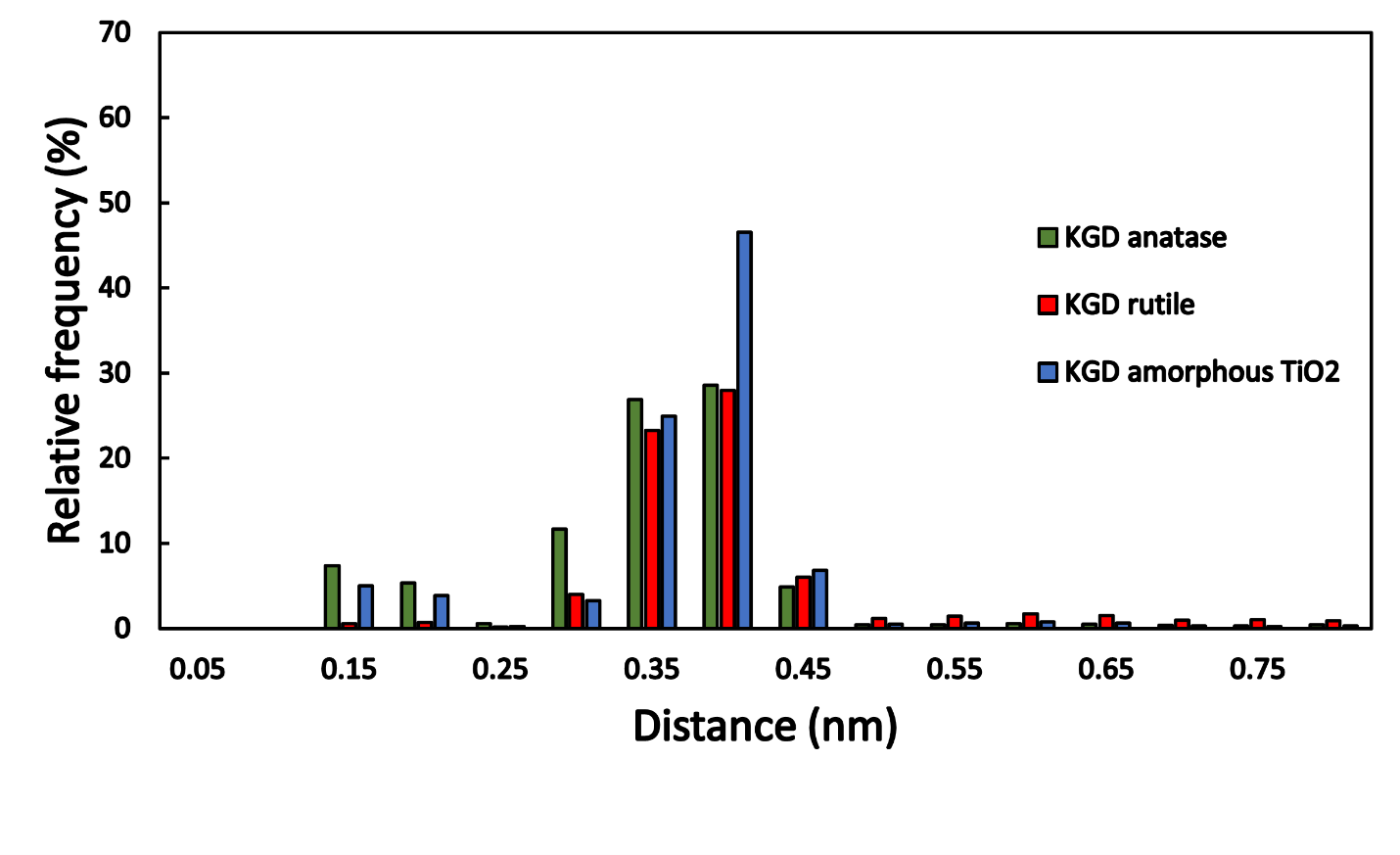

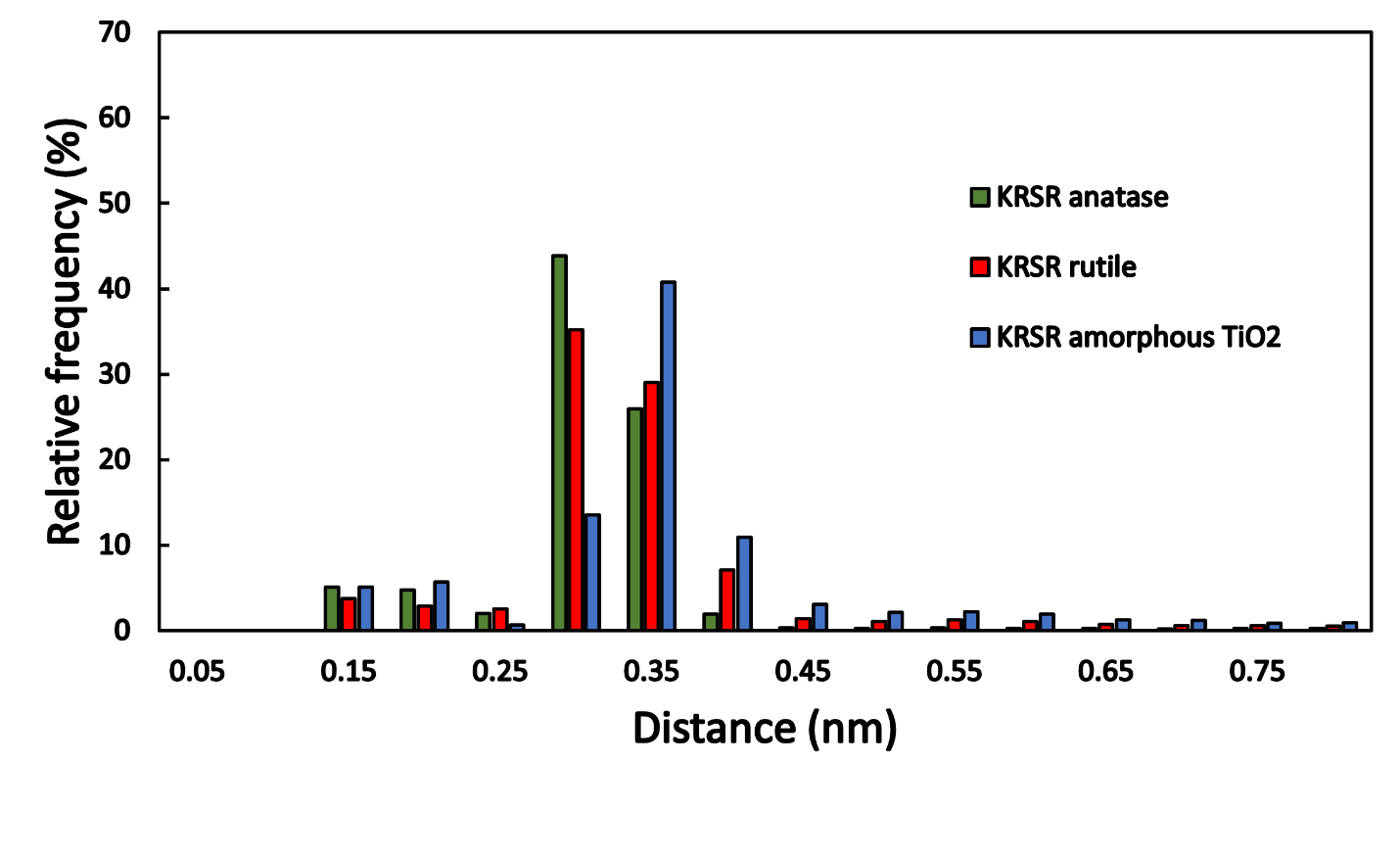

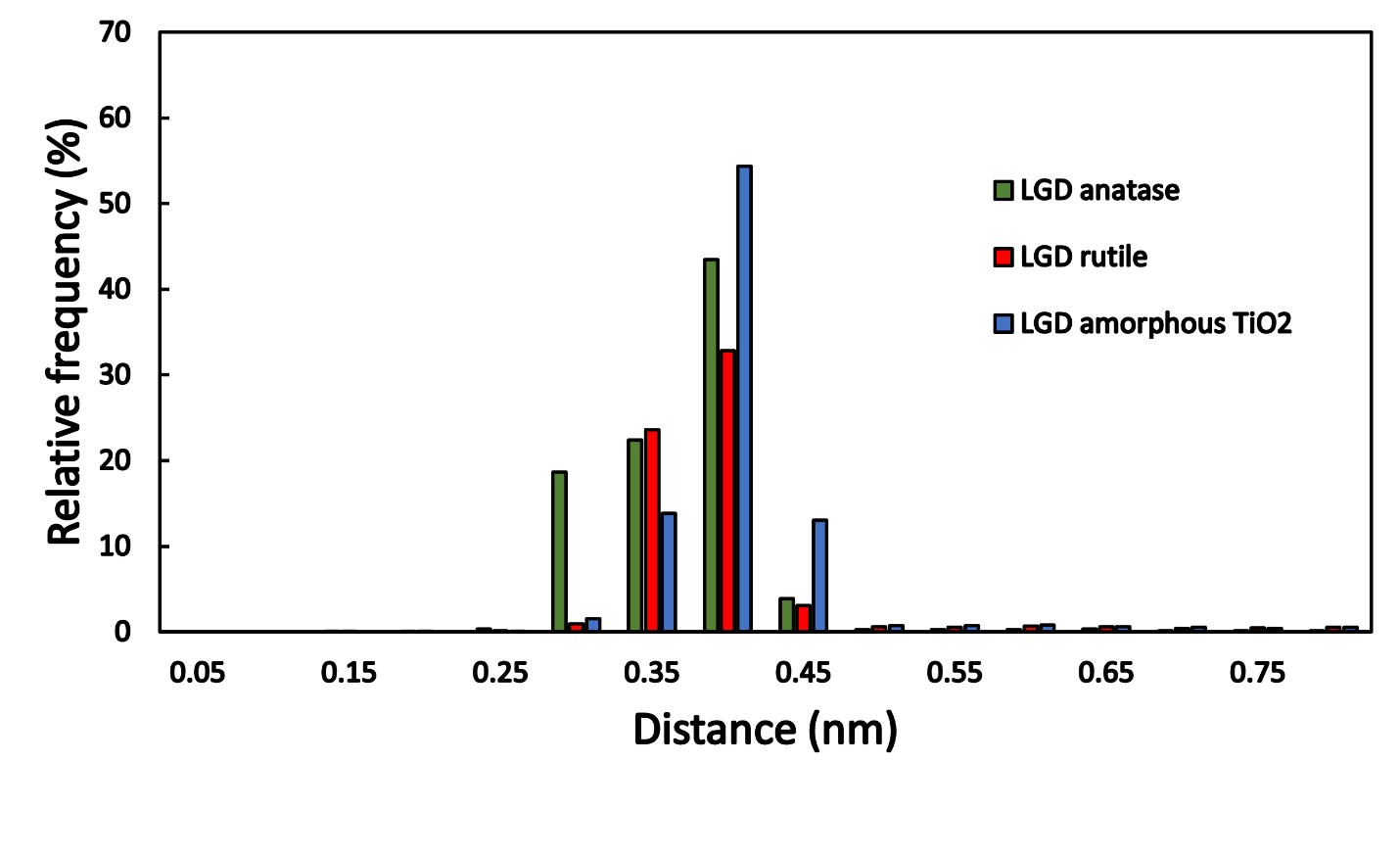
**

**Fig. A1.** Distribution of the peptide-TiO_2_ surface distances averaged for the whole simulation time. Note, that results are shown up to 0.8 nm only and molecules may spend considerable amount of time also in unbound state, where the distance is larger than this value.

The RMSD describes how much the atomic coordinates of the biomolecules change compared to the initial geometry. In the **Table A1.** The RMSD, the minimum distance and frame time values are summarized. The force-distance diagrams are displayed in **Fig. A2**, showing the pulling force acting on the distinct peptides as a function of the distance from the three different Ti surface types. There is an initial steeper phase; indicating that the adsorption forces are highest near the surface. To overcome the adsorption of the peptides to the various TiO_2_ surfaces a certain force is required. If this force is exceeded the molecules are able to move away from the surface. This distance - where the highest force values may be found – was below 0.5 nm in all cases. After the peptides begin to move away from the surface, the force values, which describe how much force is required to pull the peptides, slowly decrease as the peptides enter the water phase near the TiO_2_ surface. At distances between 0.5 – 1 nm, the adsorption force of the surface decreases considerably. At this stage, the curves show a decaying nature. After the peptides enter the bulk water phase (distance from the TiO_2_ surface > 1 nm), the pulling force is oscillating around zero. This oscillation is originated from the interaction with the surrounding water molecules.

To analyze the peptide-TiO_2_ surface interactions the peptide residue distance to the surface were determined from the simulations as well. The distances were determined separately for the starting amino acid part, N terminal and for the ending amino acid, C terminal and the results are shown as a function of simulation time on **Fig. A3**. Several different conformations are present over the 500 ns MD simulation. There are states where the N terminal interacts with the surface and the rest of the peptide is in the water phase (see e.g. KRSR on rutile surface at ~250 ns). There are conformations where the molecule spreads over the surface or forms a bridge-like state (e.g. KRSR molecule on the anatase surface at 150 ns). There are conformations, too, where mostly the C terminal interacts with the surface (e.g. KGD on amorphous surface between 100 ns and 200 ns).

|  | **Anatase TiO_2_** | | | | | | **Rutile TiO_2_** | | | | | | **Amorphous TiO_2_** | | | | | |
| --- | --- | --- | --- | --- | --- | --- | --- | --- | --- | --- | --- | --- | --- | --- | --- | --- | --- | --- |
| Peptide | KGD | KRSR | LGD | LRSR | RGD | RSR | KGD | KRSR | LGD | LRSR | RGD | RSR | KGD | KRSR | LGD | LRSR | RGD | RSR |
| RMSD (nm; mean ± SD) | 0.23 ± 0.04 | 0.34 ± 0.04 | 0.22 ± 0.03 | 0.30 ± 0.06 | 0.28 ± 0.06 | 0.27 ± 0.05 | 0.27 ± 0.05 | 0.30 ± 0.05 | 0.24 ± 0.03 | 0.31 ± 0.08 | 0.26 ± 0.06 | 0.29 ± 0.07 | 0.24 ± 0.04 | 0.30 ± 0.04 | 0.22 ± 0.04 | 0.30 ± 0.06 | 0.27 ± 0.05 | 2.22 ± 0.01 |
| Minimal distance to surface (nm) | 0.126 | 0.131 | 0.142 | 0.151 | 0.134 | 0.133 | 0.133 | 0.131 | 0.133 | 0.138 | 0.177 | 0.130 | 0.131 | 0.128 | 0.220 | 0.130 | 0.132 | 0.127 |
| Time of minimal distance (ns) | 28.54 | 149.2 | 271.0 | 479.9 | 197.5 | 113.9 | 70.48 | 472.2 | 436.5 | 285.8 | 418.4 | 243.0 | 393.6 | 34.78 | 158.7 | 56.8 | 78.0 | 491.0 |

**Table A1.** The average root-mean-square deviation of the atomic coordinates of the different peptides on the different TiO_2_ polymorphs and their standard deviation. The minimal distances of the closest atom of the peptide to the surface are also shown and the corresponding time points. The frames (atomic coordinates) corresponding to these time points were used as an initial state for the pull simulation.

**
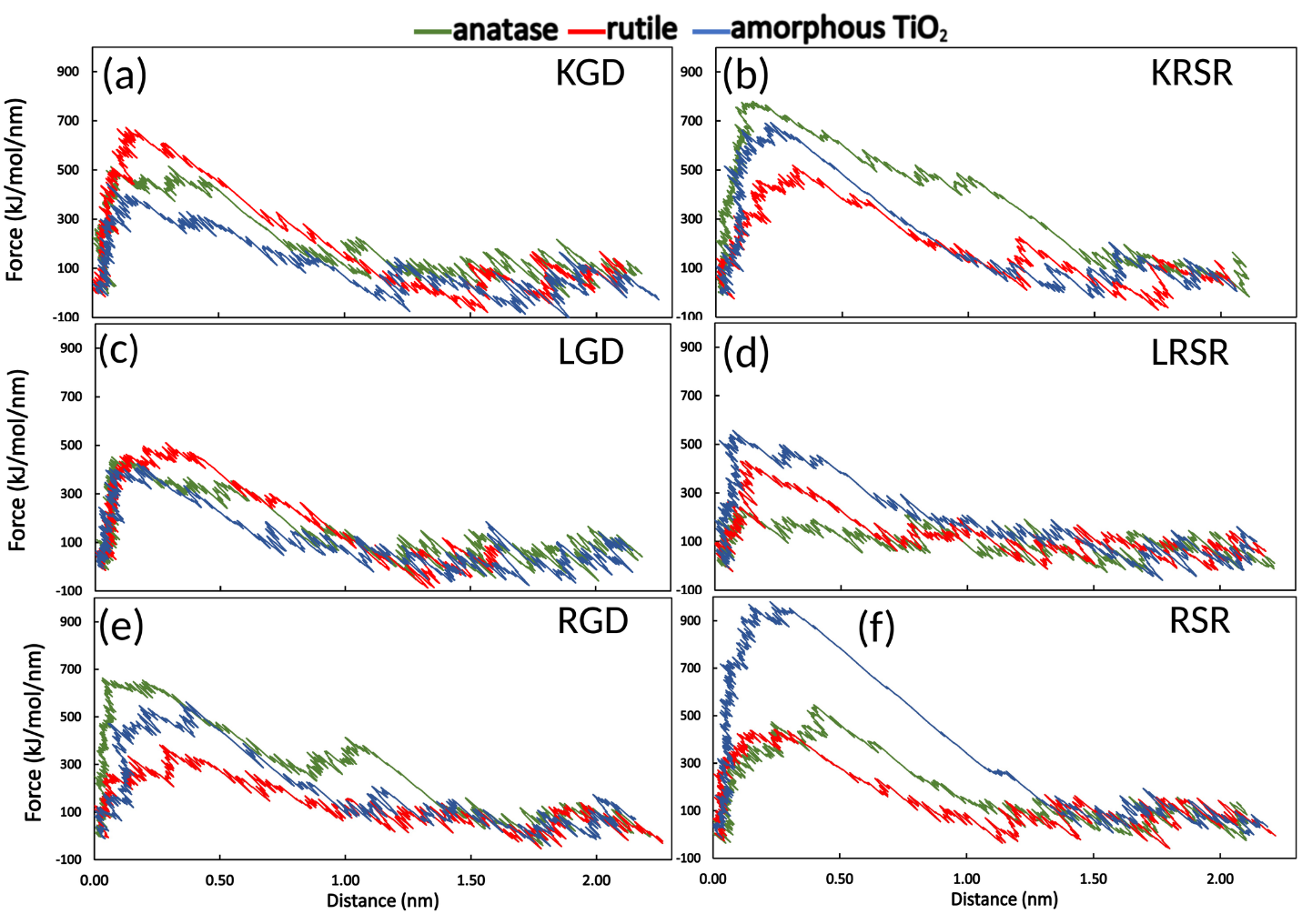
**

**Fig. A2.** The force-distance diagram shows the pulling force acting on the molecule as a function of the distance from the TiO_2_ surface.


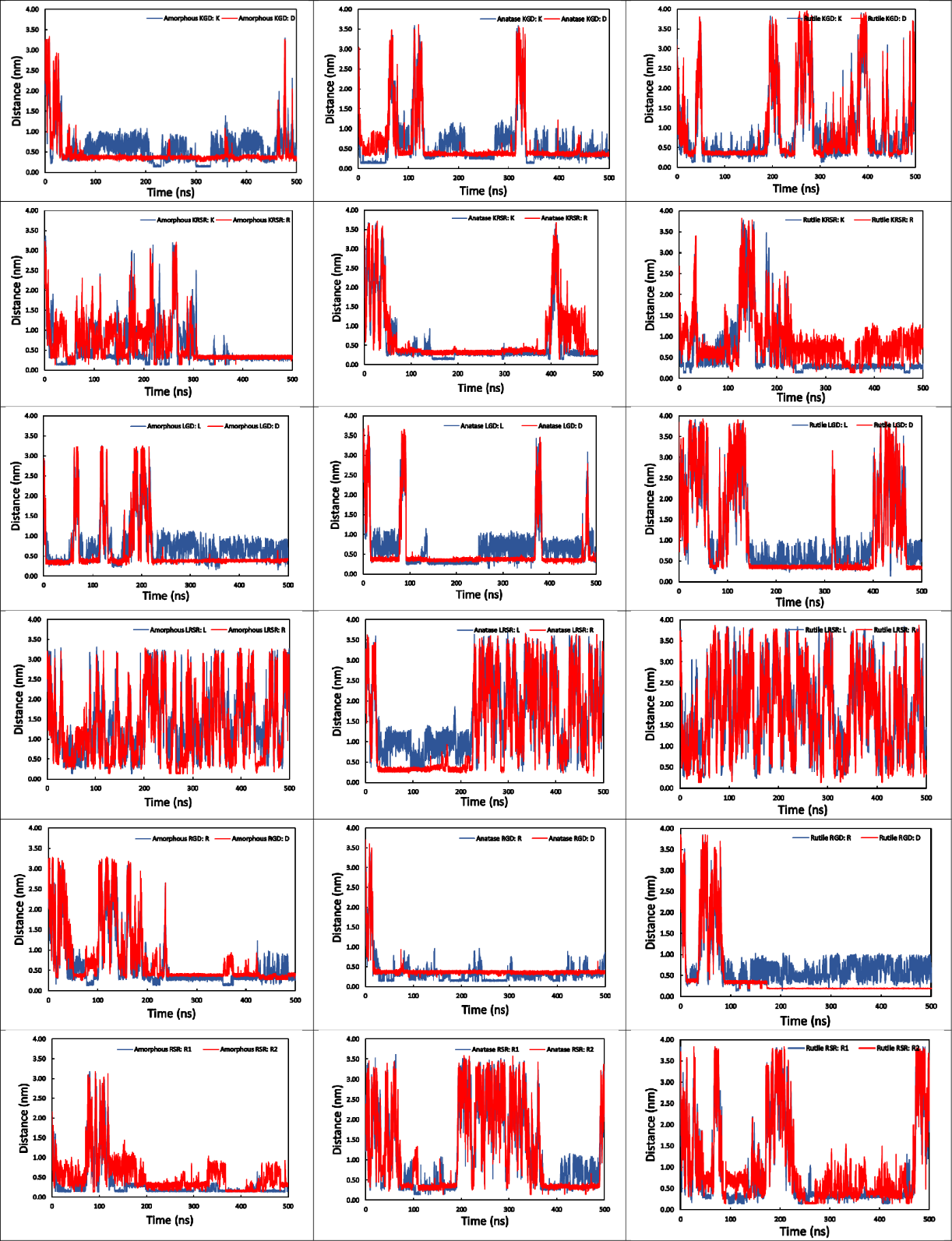


**Fig. A3** Time dependence of the distances of the N terminals (blue curves) and C terminals (red curves) of the studied biomolecules from the three kinds of TiO_2_ surfaces.
